# Learning Rat-Like Behavioral Interaction Using a Small-Scale Robotic Rat

**DOI:** 10.34133/cbsystems.0032

**Published:** 2023-06-19

**Authors:** Hongzhao Xie, Zihang Gao, Guanglu Jia, Shingo Shimoda, Qing Shi

**Affiliations:** ^1^ Intelligent Robotics Institute, School of Mechatronical Engineering, Beijing Institute of Technology, Beijing 100081, China.; ^2^ Key Laboratory of Biomimetic Robots and Systems (Beijing Institute of Technology), Ministry of Education, Beijing 100081, China.; ^3^ Nagoya University Graduate School of Medicine, Nagoya, Japan.

## Abstract

In this paper, we propose a novel method for emulating rat-like behavioral interactions in robots using reinforcement learning. Specifically, we develop a state decision method to optimize the interaction process among 6 known behavior types that have been identified in previous research on rat interactions. The novelty of our method lies in using the temporal difference (TD) algorithm to optimize the state decision process, which enables the robots to make informed decisions about their behavior choices. To assess the similarity between robot and rat behavior, we use Pearson correlation. We then use TD-*λ* to update the state value function and make state decisions based on probability. The robots execute these decisions using our dynamics-based controller. Our results demonstrate that our method can generate rat-like behaviors on both short- and long-term timescales, with interaction information entropy comparable to that between real rats. Overall, our approach shows promise for controlling robots in robot–rat interactions and highlights the potential of using reinforcement learning to develop more sophisticated robotic systems.

## Introduction

Technological advances permit the use of robots that can adapt to the situations they face and the conspecifics they encounter, or robots that learn to optimize their social performance from a set of experiences [1]. Biomimetic robots are easier to operate than real animals, and their behavior characteristics can be accurately controlled [2,3]. Through effective control methods and active guidance, these robots can interact with animals as well as observe and record their reactions [4–8]. Sometimes, biomimetic robots are placed in a group of animals to explore their behavior mechanism or to verify scientific assumptions in the process of interaction [9–12]. For example, Kopman *et al.* [6] studied the response of zebrafish to a robotic fish. The robotic fish is similar to the real zebrafish in shape and color. The experimental results show that the response of zebrafish changes with the tail-beating pattern of the robotic fish. Halloy *et al.* [9] introduced a specially designed biomimetic autonomous robot into cockroaches to study the collective decision-making behavior of cockroaches in shelter selection.

The social activities of rats have attracted the interest of many researchers, and these studies have achieved remarkable results [13,14]. Because of the advantages of biomimetic robots in animal interactions, various rat-like robots have been designed for the study of rats [15–18]. Del Angel Ortiz *et al.* [19] designed a robotic rat (e-puck) with a simple behavior pattern to explore whether the robot can interact well with actual rats and trigger their social behaviors. Heath *et al.* [20] developed a biomimetic robotic rat (Pirat), which is similar in size to the real rat. The influence of the robot on rat behaviors was tested by controlling different behaviors of the robot (frequent approach and avoidance). Sullivan *et al.* [21] designed a robot imitating the behavior of rat pups and planned the input/output cognitive architecture for the robot through a genetic algorithm to deeply understand the behavior of Norwegian rat pups.

This paper is extended from the conference version [22] by adding a state decision method for biomimetic robots to generate rat-like behavioral interactions. In our conference paper, we classified the roles of the 2 rats as an active stimulator and a passive receiver. We observed that these roles were based on 6 fundamental behaviors, namely, sniffing, exploring, walking, trotting, resting, and grooming. Each behavior involves a sequence of movements, such as head pitching, head yawing, and body pitching (Table 1). Nevertheless, to effectively conduct rat-like behavioral interactions using robots, it is essential to comprehend the mechanisms that govern the selection of behaviors. In the field of animal behavior, researchers typically employ statistical tools to analyze animal behavior data. This approach focuses on the distribution of data, rather than the connections and transitions between different behaviors. For instance, Lorbach *et al.* [23] established a behavior database by studying the distribution of mouse behavior data, which indicated that sniffing behavior is one of their preferred behaviors. Statistical tools generally only include the probability characteristics of the overall data and do not take into account the temporal characteristics of the data. Therefore, these tools are only suitable for analyzing the distribution of animal behavior and not appropriate for modeling animal behavior decision-making processes.

The decision-making modeling problems can be solved with reinforcement learning (RL). RL involves teaching intelligent agents to take action in an environment that will maximize the cumulative reward they receive. In RL, the focus is on balancing the exploration of uncharted territory with the exploitation of existing knowledge, based on observations of the environment. By using RL, intelligent agents can make decisions that are well informed and effective, even in complex and dynamic environments. For example, Kim *et al.* [24] demonstrated that intrinsically generated electroencephalography-based human feedback in RL can successfully be used to implicitly improve gesture-based robot control during human–robot interaction. Espinosa *et al.* [25] developed an efficient biologically inspired planning algorithm named TLPPO that allows the researchers to achieve mouse-level predator evasion performance with orders of magnitude less computation than a widespread algorithm for planning in the situations of partial observability that typify predator–prey interactions. Consequently, much of social robot–animal interactions can be formulated as a sequential decision-making tasks, which can be modeled by RL problems [26].

In this paper, we present a state decision method that allows robots to interact in a rat-like manner by combining known behaviors using RL. Our method begins by evaluating the similarity between the behavior of the robots and real rats using representational similarity analysis (RSA). The state accumulate reward is accordingly defined as the sum of the behavior similarity when the robots stays in the state. We then update the state value function and select actions based on the temporal difference (TD) learning algorithm. To conduct the selected actions, the robot motions are generated based on the typical characteristic movements. For the safety of the robots during behavioral interactions, we have developed wheels and torso controllers based on the robot dynamics. In what follows, the behavior similarity evaluation and the TD algorithm implementation will be described in the “RL for behavioral interaction” section, and the motion generation and control will be described in the “Robot control for behavioral interaction” section.

## Materials and Methods

### RL for behavioral interaction

**Fig. 1.**
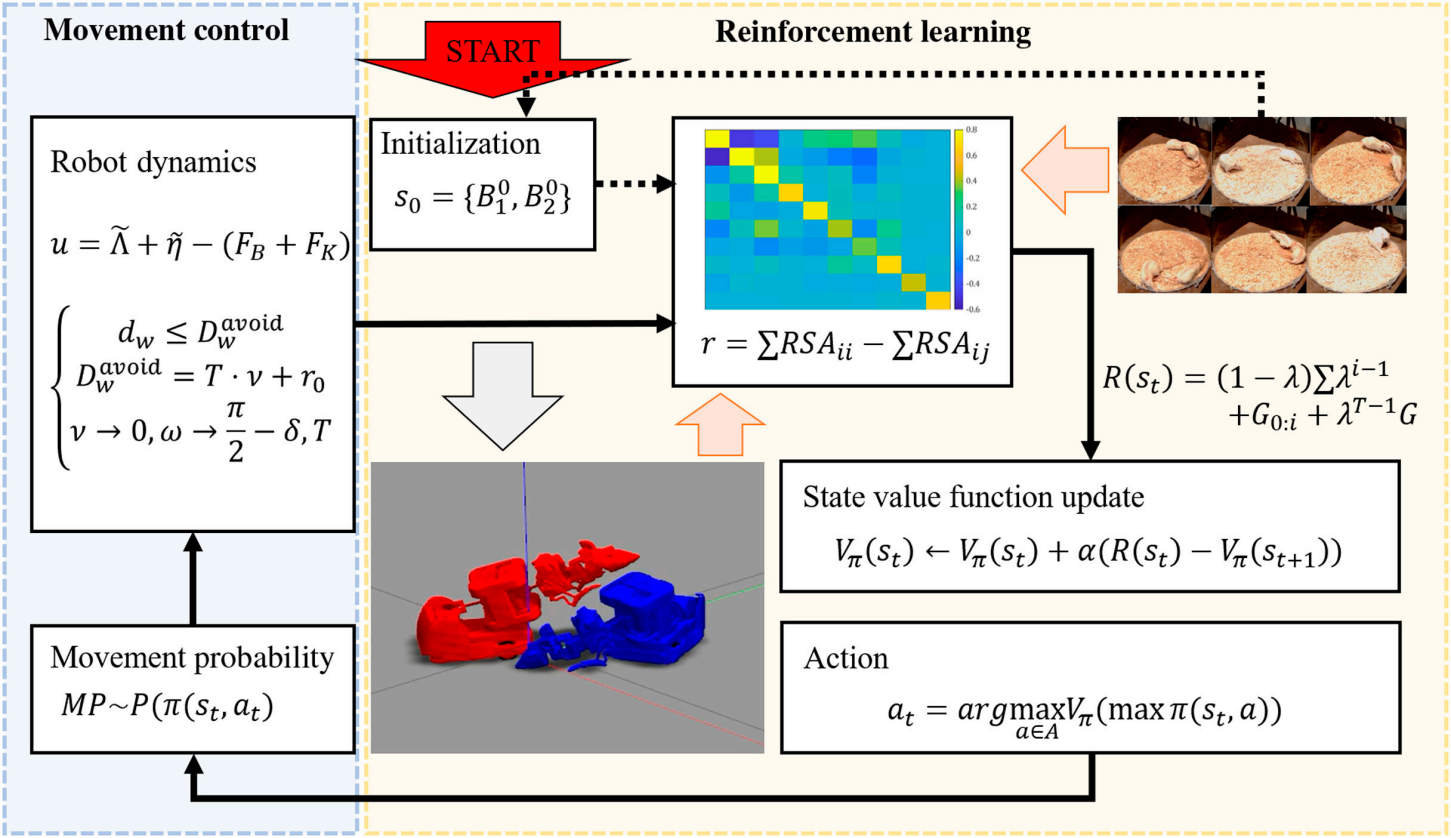
The complete process of our state decision method for generating rat-like behavioral interactions. The black solid arrows represent the different steps of the learning and control workflows. The black dashed arrows indicate the initial state initialization, which marks the beginning of our method. The red shadow arrows represent the immediate reward calculation steps, while the gray shadow arrow represents the control command given to the robots in the imulation.

The framework of our state decision method is depicted in Fig. 1. The method starts by initializing the robot’s state to match the real rat’s interaction. It then evaluates the similarity between the robot’s behavior and that of the rat and updates the state value function using RL to generate an appropriate action. The resulting action is translated into a robot control command through the movement generation and dynamics control module. Subsequently, the robots in the simulation execute the command, and the method moves on to the next step. This process enables the robot to make decisions that closely resemble those of the rat, providing a powerful tool for studying and simulating complex behaviors in rodents.

**Table 1.** Movement and behavior of rats.

Movement	Symbol	Behavior	Symbol
Head pitching	*M*_1_	Sniffing	*B*_1_
Head yawing	*M*_2_	Exploring	*B*_2_
Body pitching	*M*_3_	Walking	*B*_3_
Body yawing	*M*_4_	Trotting	*B*_4_
Going straight	*M*_5_	Resting	*B*_5_
Turning	*M*_6_	Grooming	*B*_6_
Staying	*M*_7_		
Forelimb swing	*M*_8_		

#### Preliminaries

We consider behavioral interaction as a Markov decision process (MDP), which is defined as a 4-tuple $M=\left\{S,A,P_{a},R_{a}\right\}$, where S is a set of states called the state space, A is a set of possible actions the robot can take, $P_{a}\left(s,s^{\prime }\right)$ is the probability that action *a* in state *s* at time step *t* will lead to state *s*^′^ at time step *t* + 1, and $R_{a}\left(s,s^{\prime }\right)$ is the immediate reward (or expected immediate reward) received after transitioning from state *s* to state *s*^′′^, due to action *a* [27]. We construct our state space based on the behavior being performed by the 2 rats (denoted by rat 1 and rat 2). Every state is the combination *s* = (*B*_1_, *B*_2_). Therefore, the size of the state space is 16. The behavior of a rat under state *s* is described as functions of a 3-tuple {*T*, *f*, *ϕ*}*_s_*, where *T* is the time duration, *f* is the motion or behavior frequency, and *ϕ* is the amplitude of the motion of the state *s*. Accordingly, the action space contains the transition between each state in the state space. Our aim is to find the optimal policy at time *t* to reach the point where the accumulated interaction information entropy is less than elsewhere.

$$\begin{array}{c} \pi^{*}\left(s,a\right)=\text{arg}\underset{a\in A}{\text{min}}\int I^{t}\left(s∼\pi \left(s,a\right)\right)\mathrm{dt}\\ s.\;t.I^{t}\left(s\right)=\sum_{B_{1}^{t+1},B_{2}^{t},s_{2}^{t+1}}p\left(B_{1}^{t+1},\;B_{2}^{t},\;B_{2}^{t+1}\right){\text{log}}_{2}\left(\frac{p\left(B_{2}^{t+1}|B_{1}^{t+1}\right),p\left(B_{2}^{t}\right)}{p\left(B_{2}^{t+1}|B_{2}^{t}\right)}\right) \end{array}$$ (1)

where *π*^∗^ is the optimal policy given states of the 2 rats *s*_1_, *s*_2_ and action *s* in the format of probability density function, and *I* is the interaction information entropy between rats. The details of the interaction information entropy can be found in [22,28].

#### Behavior similarity evaluation

In MDP, the immediate reward should be given as quickly as possible to the agent so that it can take action at once to accommodate itself to the environment. But the calculation of the value of the interaction information entropy is heavy computation. That means an immediate evaluation method to measure the actions in interaction is required to speed up the decision-making. To this end, we use the well-validated method of representational similarity analysis (RSA) [29] at a short timescale to calculate the reward.

Let Θ denote the animal motion description variables.

$$\Theta ={\left[\theta_{1},\theta_{2},\cdots ,\theta_{7},\nu ,\omega \right]}^{T}$$ (2)

where *θ_i_*, (*i* = 1, 2, ⋯, 7) represents the *i*th joint displacements of the animal and *ν*, *ω* represents the linear and the angular velocity of the animal with respect to the world frame. It can be proved that the expected interaction information entropy is the function of motion description variables at time *t*, with no need of future knowledge (Section S1). Therefore, it is reasonable to use Θ to calculate the current immediate reward. As multi-channel measures of motions are quantitatively related to each other and to computational theory and behavior by comparing representational dissimilarity matrices, RSA can be seamlessly applied in this context [30].

**Fig. 2.**
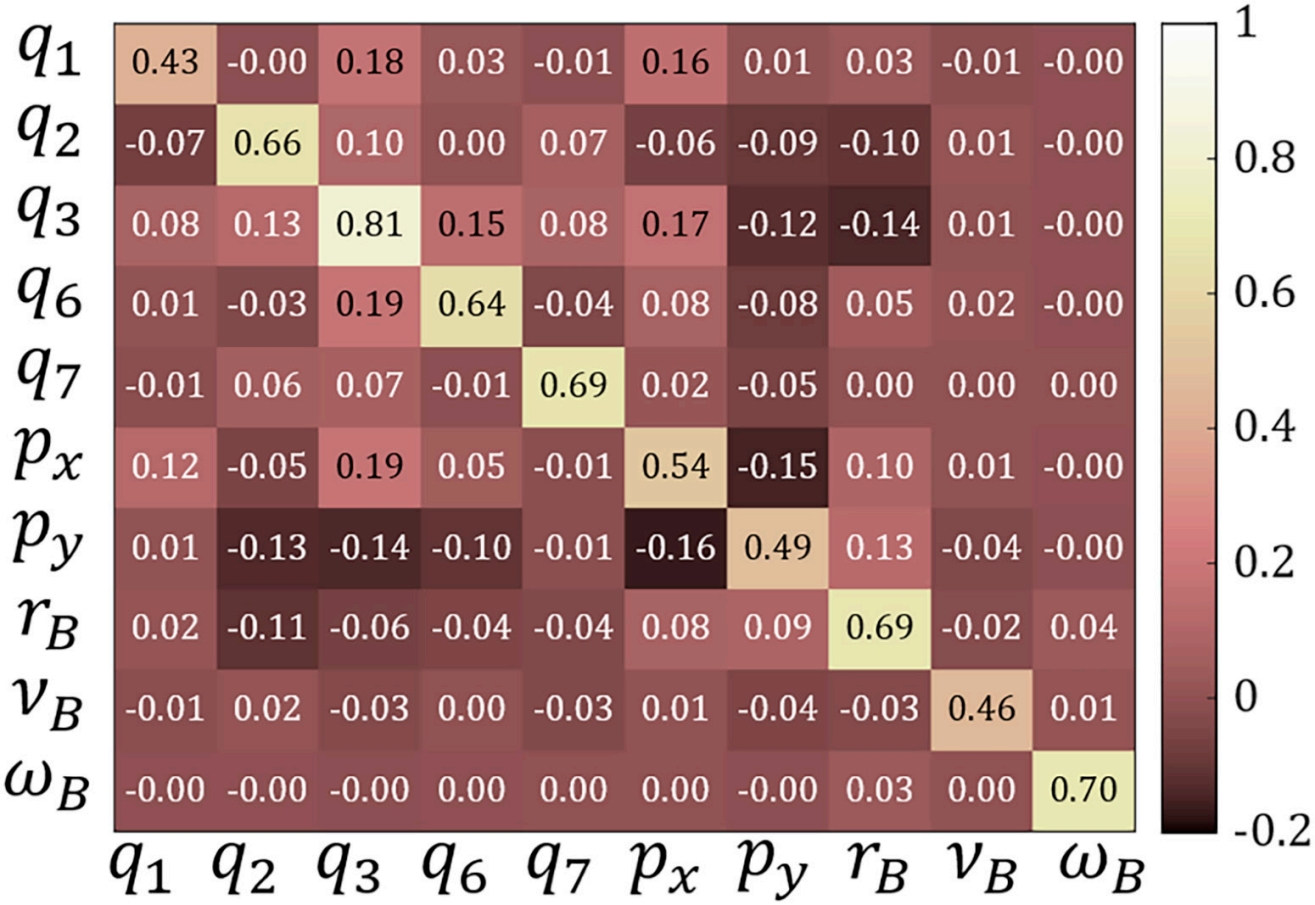
The kinematic similarity matrix generated using RSA. The horizontal axis represents the joint displacements of the real rats. The vertical axis represents the joint displacements of the policy-generated data.

To apply RSA, we first calculate the Pearson correlation of the motions between the policy-generated data and real rats’ motion data. Then, we calculate the kinematic similarity matrix at the time step as *RSA* (Fig. 2) with the size of *l* × *l*. We then convert the matrix into a scalar as the immediate reward by

$$r=\sum_{i=1}^{l}{\mathrm{RSA}}_{\mathrm{ii}}-m\sum_{j,k=1,j\neq k}^{j,k=l}\left|{\mathrm{RSA}}_{\mathrm{jk}}\right|$$ (3)

where RSA*_ij_* represents the elements at the *i*th row and the *j*th column of RSA matrix, and *m* is a constant coefficient. It means that the immediate reward will increase as the diagonal elements of RSA increase and other elements of RSA decrease.

#### TD learning-based state decision

In our behavioral model, the rats will stay in one state in a good many steps, which can be modelled in the following formula:

$$s_{t+1}=\left\{\begin{array}{cc} s_{t}, & 0<t<T_{s}\\ \pi \left(s_{t},a_{t}\right), & \;t=T_{s} \end{array}\right.$$ (4)

It means that our policy generates new actions only when the rats finish executing one state. At the same time, the policy will be updated. Therefore, we are expected to solve the interaction MDP at the end of each state.

Due to the fact that it is hard to know exactly the probability of state transition of the rat interaction model, we can not solve the interaction MDP directly using the Bellman equation (Eq. 5)

$$V_{\pi }\left(s\right)=\sum_{a\in A}\pi \left(a|s\right)\left(R_{s}^{a}+\gamma \sum_{s'\in S}P_{ss^{\prime }}^{a}V_{\pi }\left(s^{\;'}\right)\right)$$ (5)

where *V_π_* is the state value function of MDP under the policy *π* and *γ* is the discount coefficient. TD learning refers to a class of model-free RL methods, which learn by bootstrapping from the current estimate of the value function. These methods sample from the environment, like Monte Carlo methods, and perform updates based on current estimates, like dynamic programming methods. Using TD learning algorithm, the accumulated reward of the state can be given in

$$R\left(s\right)=G_{0}^{\lambda }=\left(1-\lambda \right)\sum_{i=1}^{T-1}\lambda^{i-1}G_{0:i}+\lambda^{T-1}G_{0}$$ (6)

The detail of the accumulate reward can be found in Section S2.

Using the accumulate rewarjointhe state, we can then update the state value function using Eq. 7. The action can be generated using Eq. 8.

$$V_{\pi }\left(s_{t}\right)\leftarrow V_{\pi }\left(s_{t}\right)+\alpha \left(R\left(s_{t}\right)-V_{\pi }\left(s_{t+1}\right)\right)$$ (7)

$$a_{t}=\text{arg}\underset{a\in A}{\text{max}}V_{\pi }\left(\text{max}\pi \left(s_{t},a\right)\right)$$ (8)

### Robot control for behavioral interaction

#### Robotic rat platform

**Fig. 3.**
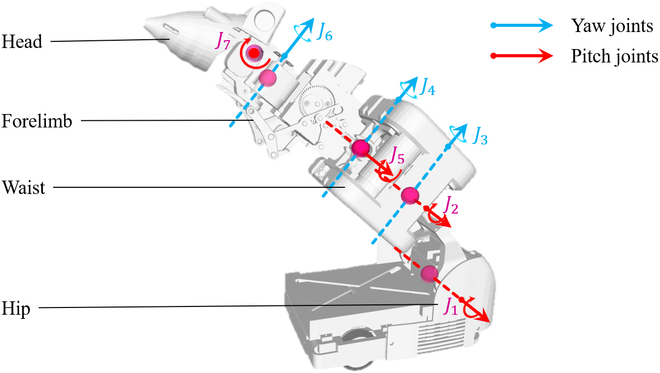
The robotic rat and its coordinate system.

The prototype of our recently developed robotic rat is shown in Fig. 3. The robot is mainly composed of the head, forelimb, waist, and hip. The head and hip joints are driven by servo motors, the waist joints and wheels are driven by DC motors, and the forelimb is driven by a micro-deceleration stepper motor. Its shape and size are similar to those of actual rats, with a total mass of approximately 400 g. The spinal joints of the robot correspond to the key movement joints of rats. The head pitching and yaw movements (*M*_1_, *M*_2_) are performed by *J*_7_ and *J*_6_, respectively; the body pitching movement *M*_3_ is performed by *J*_1_, *J*_2_, and *J*_5_; the body yaw movement (*M*_4_) is performed by *J*_3_ and *J*_4_; the straight movement (*M*_5_) is performed by the driving wheels (*J_WL_*, *J_WR_*); the turning movement (*M*_6_) is performed by the yaw joints compound driving wheels (*J*_3_, *J*_4_, *J*_6_, *J_WL_*, *J_WR_*); and the forelimb swing movement (*M*_8_) is performed by *J_F_*.

#### Characteristic movements

Our previous study introduced a hierarchical state–behavior–motion model parameterizing the behavior using a probabilistic model [28]. The work provided the value of the probability of each movement in different behaviors (Table 2.). Using the result, we will modulate the movements of the robots according to the probabilistic model.

**Table 2.** Movement probability of rats in different behaviors.

Behavior	Sniffing	Exploring	Walking	Trotting	Resting	Grooming
Head pitching	1	0.33	0	0	0	0.5
Head yawing	0	0.33	0	0	0	0
Body pitching	0	0.25	0	0	0	0
Body yawing	0	0.09	0	0	0	0
Going straight	0	0	0.5	0.5	0	0
Turning	0	0	0.3	0.5	0	0
Staying	0	0	0.2	0	1	0
Forelimb swing	0	0	0	0	0	0.5

To make the robot achieve our actions in different states, we extracted the characteristic parameters for each movement combination. These parameters determine the amplitude, average speed, frequency, and duration of rat movements. In our previous work, we proved that the robot can complete the movement of rats with a high biomimicry degree, and the trajectory of each joint of the robot can be expressed by the above characteristic parameters [31]. All of these movements have their frequency and duration. Besides, each movement has its independent characteristic parameter indicating its amplitude or average speed. Thus, we can denote the movements of a robot in a 3-tuple {*T*, *f*, *ϕ*}. In our behavior model described in the “RL for behavioral interaction” section, the 2 robots in interaction should start and end their movements at the same time. Hence, the movement parameters of the 2 robots are a 5-tuple {*T*, *f*_1_, *f*_2_, *ϕ*_1_, *ϕ*_2_}, where the subscripts of *f* and *ϕ* represent different robots. To simplify the training process, we use the typical value of each parameter to control the movements (Table 3). These values are summarized from more than 3,000 movements of real rats [22].

**Table 3.** Typical movement parameters.

Movements	Duration (*T*)	Frequency (*f*)	Amplitude (*ϕ*)
Head pitching	2	3	0.6
Head yawing	2	1	0.4
Body pitching	3	1	0.5
Body yawing	2.5	1	0.4
Going straight	1	–	0.3
Turning	2	–	*π*/2
Staying	1	–	–
Forelimb swing	1	–	–

#### Control framework

In behavioral interaction, there are a lot of contacts and collisions between 2 rats, especially when they are sniffing or grooming each other. To make our robot behave in a stable and proper manner in these circumstances, we implement an impedance control algorithm for the robot torso part. The torso part containing 8 links can be viewed as a chain robotic arm with its head link as end effector. In our work of interaction, the input force command *u* of the robot satisfies the following equation:

$$u=\tilde{\Lambda }\left(K_{p}x+K_{d}\dot{x}\right)\ddot{q}+\tilde{\eta }\left(q,\dot{q}\right)-\left(F_{B}\dot{x}+F_{K}x\right)$$ (9)

where *x* is the displacement of the joint-space configuration coordinates from desired positions; *F_M_*, *F_B_*, and *F_K_* are the positive-definite virtual mass, damping, and stiffness matrices to be simulated by the robot torso, respetively; *f*_ext_ is a force applied by other subjects, perhaps another robot; and $\left\{\tilde{\Lambda },\tilde{\eta }\right\}$ is the joint-space dynamic model [32].

**Fig. 4.**
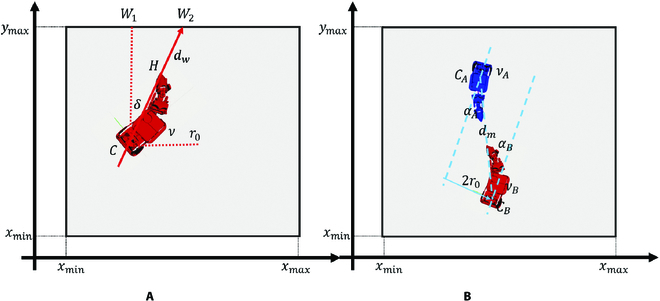
Obstacle avoidance analysis. (A) Avoid collision with boundary (wall). (B) Avoid collision between robots.

For the security of robots, we considered the problem of robot collision avoidance, as shown in Fig. 4. Figure 4A shows the process of the robot avoiding collision with the boundary (wall). The condition of obstacle avoidance and the configuration of robot movement parameters are shown in Eq. 10. Figure 4B shows the process of avoiding collisions between robots. The condition for obstacle avoidance and the configuration of robot movement parameters are shown in Eq. 11.

$$\left\{\begin{array}{c} d_{w}\leq D_{w}^{\text{avoid}}\\ D_{w}^{\text{avoid}}=T_{\text{control}}\cdot \nu +r_{0}\\ \nu \to 0,\alpha =\pi /2-\delta ,T_{\text{control}} \end{array}\right.$$ (10)

$$\begin{array}{c} \left\{\begin{array}{c} d_{m}\leq D_{m}^{\text{avoid}}\\ D_{m}^{\text{avoid}}=T_{\text{control}}\cdot \nu_{m}+2r_{0}\\ \;\nu_{A}\to 0,\alpha_{A},T_{\text{control}}\\ \;\nu_{B}\to 0,\alpha_{B},T_{\text{control}} \end{array}\right. \end{array}$$ (11)

## Results

### Experimental setup

In this section, we present the experimental evaluation of the proposed method. Due to the fact that the training and validation process requires huge amount of computation, we evaluate our algorithm in our simulation system for behavioral interaction [33]. The simulation system is developed on Robot Operating System (ROS) with gazebo simulator in Ubuntu 20.04. Our simulation computer’s configuration is 32GB RAM and NVIDIA GeForce RTX 3070 graphic card. The behavior and movement data of real rats are acquired by the OptiTrack motion capture system [34]. The total amount of the data is about 150,000 frames, and 80% of the data is used for training, while 20% is used for validation. To take advantage of the data using our robot, the conversion from rat data to robot joints is adopted before the experiment, which was described in [33].

**Fig. 5.**
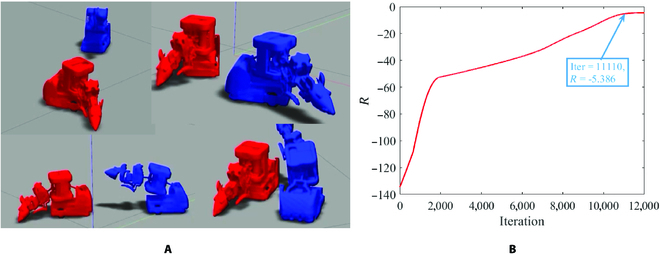
The simulation experiments between 2 robots. (A) Example screenshots of robot behavioral interaction in rat-like manner. (B) Immediate reward versus iteration during the training phase.

In the simulation, we construct 2 robots in the gazebo, each of whom represents one rat in behavioral interaction. The 2 robots are both controlled under our policy framework, but they are trained respectively. The behavior screenshots of the robots in the simulation are provided in Fig. 5A. It takes about 12,000 iterations to train our policy before the reward no longer ascends (Fig. 5B). After the policy converges, we adopt 2 main metrics: (a) the behavior distribution of the robots and real rats, (b) the behavior similarity between the real rats and the robots, and (b) the interaction information entropy of the robot behavioral interaction to assess our method. The latter part of this section will analyze these 2 indicators.

### Behavior similarity

**Fig. 6.**
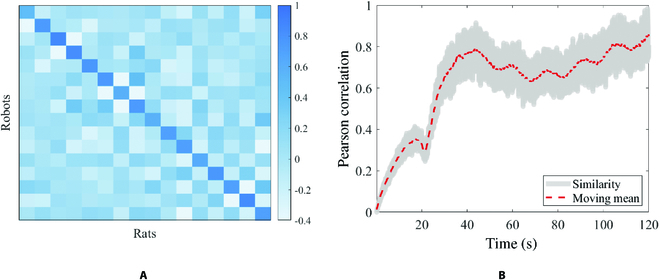
Behavior similarity between the robots and the rats. (A) Long-timescale similarity matrix. (B) Short-timescale similarity versus time.

To confirm that our method is able to generate rat-like behaviors, we use RSA to evaluate the behavior similarity between the robots and the rats. A challenge in understanding the behavior is that it can be described at different timescales. Hence, we first calculate the kinematic similarity matrix across the whole validation set, which stands for long timescales. Then, we calculate the Pearson correlation coefficient between the robots’ and the rats’ behavior data in the validation set with a 240-frame (1 s) smooth window, which is considered a short timescale. The results are presented in Fig. 6.

In Fig. 6A, the behavior similarity is higher in those same kinematic parameters than in others. Figure 6B shows that the short-timescale similarity is at a low position during the first 30 s. But the robots could adjust their movements quickly, and the similarity becomes higher after 30 s.

### Interaction information entropy

**Fig. 7.**
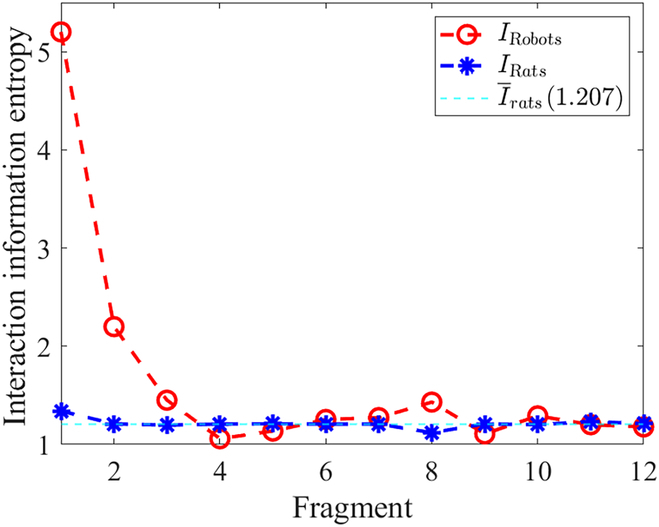
The interaction information entropy of the robots and the rats in different time fragments.

Our goal is to find a policy that reduces the interaction information entropy between 2 robots. To compare the value at different times, we cut the data into 12 fragments, and each fragment lasts for 10 s. Then, we calculate the accumulated interaction information entropy *I*_robots_ and *I*_rats_ in these fragments. The results are presented in Fig. 7. It shows that although *I*_robots_ is higher at the first 2 fragments, it can reduce close to *I*_rats_ after *t* = 30 s.

## Discussion

This paper aims to learn the optimal policy for robots in generating rat-like behavioral interaction using the predefined typical behavior states. In order to combine these behaviors, we proposed a state decision method based on the TD algorithm. It updates the state value function and decides the action based on behavior similarity between the robots and the rats.

After testing in simulation, we found that the distributions of the robot joint movements are similar with those of the real rats. The behavior similarity will increase after the robots adjusted themselves to the interaction context in about 30 s, which means our method is generalized. It means that the robots behave like real rats over a long timescale. Furthermore, the interaction information entropy of the robots is close to that of the real rats. The fact indicates that our method is able to generate optimal state decision by combining the known behaviors with a few adjustments.

The deficiency of this study is that in the interactive control of robots, the contacting state is not considered exactly. Although an impedance control framework is developed, how the contacts of the rats will influence their behaviors and further decision choice remains uncertain. In intimate interaction, the animal touch is very popular and will convey plentiful information about their feelings and emotions, which can be important factors of the interaction. In future work, we will plan to improve our state decision by integrating the animal contact and collision model.

Another deficiency is that to reduce the computation when training the method, we modulated the robot movements by some typical parameters. However, this approach harms the diversity of the robot’s behaviors. The potential behaviors of the robots become less than those of the animals. Solving the problem may require knowledge about probability trajectory generation and motion planning. We will plan to work on this issue in our future work.

In this study, the experiments are implemented in a simulation system. Transforming the method from the virtual environment to reality requires many hardware-related works including motion control, robot vision, and remote communication. In the future, we will focus on these works.

## Data Availability

Data are available upon reasonable request.

## Supplementary Materials

Supplementary 1 Sections S1 and S2

## References

[1] Tim L, Gebhardt GHW, Bierbach D, Romanczuk P, Musiolek L, Hafner VV, Krause J. Animal-in-the-loop: Using interactive robotic conspecifics to study social behavior in animal groups. Annu Rev Control Robot Auton Syst. 2020;4:487–508.

[2] Abdai J, Miklósi Á. Poking the future: When should we expect that animal-robot interaction becomes a routine method in the study of behavior? Anim Behav Cogn. 2018;5(4):321–325.

[3] Yeager J, Wooten C, Summers K. A new technique for the production of large numbers of clay models for field studies of predation. Herpetol Rev. 2011;42(3):357–359.

[4] Son J-H, Ahn H-S. A robot learns how to entice an insect. IEEE Intell Syst. 2015;30(4):54–63.

[5] Taylor RC, Klein B, Stein J, Ryan MJ. Faux frogs: Multimodal signalling and the value of robotics in animal behaviour. Anim Behav. 2008;76(3):1089–1097.

[6] Kopman V, Laut J, Polverino G, Porfiri M. Closed-loop control of zebrafish response using a bioinspired robotic-fish in a preference test. J R Soc Interface. 2013;10:20120540. 23152102 10.1098/rsif.2012.0540 PMC3565779

[7] Partan S, Larco C, Owens M. Wild tree squirrels respond with multisensory enhancement to conspecific robot alarm behaviour. Anim Behav. 2009;77:1127–1135.

[8] Landgraf T, Oertel M, Rhiel D, Rojas R. A biomimetic honeybee robot for the analysis of the honeybee dance communication system. Paper presented at IEEE: Propceedings of the 2010 IEEE/RSJ International Conference on Intelligent Robots and Systems; Taipei, Taiwan; 2010 October 18–22; pp. 3097–3102.

[9] Halloy J, Sempo G, Caprari G, Rivault C, Asadpour M, Tâche F, Saïd I, Durier V, Canonge S, Amé JM, et al. Social integration of robots into groups of cockroaches to control self-organized choices. Science. 2007;318(5853):1155–1158. 18006751 10.1126/science.1144259

[10] Ward A, Sumpter D, Couzin I, Hart P, Krause J. Quorum decision-making facilitates information transfer in fish shoals. Proc Natl Acad Sci USA. 2008;105:6948–6953. 18474860 10.1073/pnas.0710344105 PMC2383955

[11] Gribovskiy A, Halloy J, Deneubourg J-L, Mondada F. Designing a socially integrated mobile robot for ethological research. Robot Auton Syst. 2018;103:42–55.

[12] Faria J, Dyer JRG, Clément RO, Couzin ID, Holt N, Ward AJW, Waters D, Krause J. A novel method for investigating the collective behaviour of fish: Introducing ‘robofish’. Behav Ecol Sociobiol. 2010;64:1211–1218.

[13] Felix-Ortiz A, Burgos-Robles A, Bhagat N, Leppla C, Tye K. Bidirectional modulation of anxiety-related and social behaviors by amygdala projections to the medial prefrontal cortex. Neuroscience. 2015;321:197–209. 26204817 10.1016/j.neuroscience.2015.07.041 PMC4721937

[14] Weiss O, Segev E, Eilam D. “Shall two walk together except they be agreed?” Spatial behavior in rat dyads. Anim Cogn. 2014;18(1):39–51. 24958542 10.1007/s10071-014-0775-7

[15] Lucas P, Walter F. Design of a biomimetic rodent robot. 2018.

[16] Shi Q, Ishii H, Kinoshita S, Takanishi A, Okabayashi S, Iida N, Kimura H, Shibata S. Modulation of rat behaviour by using a rat-like robot. Bioinspir Biomim. 2013;8(4): 046002. 24091776 10.1088/1748-3182/8/4/046002

[17] Shi Q, Ishii H, Tanaka K, Sugahara Y, Takanishi A, Okabayashi S, Huang Q, Fukuda T. Behavior modulation of rats to a robotic rat in multi-rat interaction. Bioinspir Biomim. 2015;10(5): 056011. 26414400 10.1088/1748-3190/10/5/056011

[18] Shi Q, Gao J, Wang S, Quan X, Jia G, Huang Q, Fukuda T. Development of a small-sized quadruped robotic rat capable of multimodal motions. IEEE Trans Robot. 2022;38(5):3027–3043.

[19] del Angel Ortiz R, Contreras CM, Gutiérrez-Garcia AG, González MFM. Social interaction test between a rat and a robot: A pilot study. Int J Adv Robot Syst. 2016;13(1):4.

[20] Heath S, Ramirez-Brinez CA, Arnold JT, Olsson O, Taufatofua J, Pounds P, Wiles J, Leonardis E, Gygi E, Leija E, et al. PiRat: An autonomous framework for studying social behaviour in rats and robots. Paper presented at IEEE: Proceedings of the 2018 IEEE/RSJ International Conference on Intelligent Robots and Systems IROS; 2018 October 1–5; Madrid, Spain; pp. 7601–7608. 10.1109/iros.2018.8594060 PMC8492938 34621592

[21] Sullivan C, Loughlin R, Schank JC, Joshi SS. Genetic algorithms produce individual robotic rat pup behaviors that match norway rat pup behaviors at multiple scales. Artif Life Robot. 2015;20(2):93–102.

[22] Gao Z, Jia G, Xie H, Guo X, Fukuda T, Shi Q. Learning rat-like behavioral interaction using a small-scale robotic rat. Paper presented at: 12th International Conference on CYBER Technology in Automation, Control, and Intelligent Systems (CYBER); 2022 July 27–31; Baishan, China; pp. 701–706.

[23] Lorbach M, Kyriakou EI, Poppe R, van Dam EA, Noldus LPJJ, Veltkamp RC. Learning to recognize rat social behavior: Novel dataset and cross-dataset application. J Neurosci Methods. 2018;300:166–172. 28495372 10.1016/j.jneumeth.2017.05.006

[24] Kim SK, Kirchner EA, Stefes A, Kirchner F. Intrinsic interactive reinforcement learning—Using error-related potentials for real world human-robot interaction. Sci Rep. 2017;7:17562. 29242555 10.1038/s41598-017-17682-7 PMC5730605

[25] Espinosa G, Wink GE, Lai AT, Dombeck DA. M. A. MacIver. Achieving mouse-level strategic evasion performance using real-time computational planning. ArXiv. 2022. https://arxiv.org/abs/2211.02700

[26] Akalin N, Loutfi A. Reinforcement learning approaches in social robotics. Sensors. 2021;21(4): Article 1292. 33670257 10.3390/s21041292 PMC7918897

[27] Littman M. *Markov decision processes*. Oxford (UK): Pergamon; 2001. p. 9240–9242.

[28] Gao Z, Jia G, Xie H, Huang Q, Fukuda T, Shi Q. Learning rat-like behavior for a small-scale biomimetic robot. Engineering. 2022;17:232–243.

[29] Kriegeskorte N, Diedrichsen J. Peeling the onion of brain representations. Annu Rev Neurosci. 2019;42(1):407–432. 31283895 10.1146/annurev-neuro-080317-061906

[30] Kriegeskorte N, Mur M, Bandettini P. Representational similarity analysis – connecting the branches of systems neuroscience. Front Syst Neurosci. 2008;2: Article 4. 10.3389/neuro.06.004.2008 PMC2605405 19104670

[31] Shi Q, Gao Z, Jia G, Li C, Huang Q, Ishii H, Takanishi A, Fukuda T. Implementing rat-like motion for a small-sized biomimetic robot based on extraction of key movement joints. IEEE Trans Robot. 2021;37(3):747–762.

[32] Keemink AQ, van der Kooij H, Stienen AH. Admittance control for physical human-robot interaction. Int J Rob Res. 2018;37(11):1421–1444.

[33] Xie H, Jia G, al-Khulaqui M, Gao Z, Guo X, Fukuda T, Shi Q. A motion generation strategy of robotic rat using imitation learning for behavioral interaction. IEEE Robot Autom Lett. 2022;7(3):7351–7358.

[34] NaturalPoint, Motion capture systems—optitrack webpage. optitrack.com

