# Supplementary material for: Learning Rat-Like Behavioral Interaction Using a Small-Scale Robotic Rat
Source: Cyborg Bionic Syst. 2023 Jun 19;4:0032. doi: 10.34133/cbsystems.0032 (PMC10278959; doi:10.34133/cbsystems.0032)
Supplement: Supplementary 1 — Sections S1 and S2 [file cbsystems.0032.f1.docx]

Supplementary Materials

Learning Rat-Like Behavioral Interaction Using a Small-Scale Robotic Rat

Hongzhao Xie^1,2^, Zihang Gao^1,2^, Guanglu Jia^1,2^, Qing Shi^1,2,*^.

Affiliations

1. Intelligent Robotics Institute, School of Mechatronical Engineering, Beijing Institute of Technology, Beijing 100081, China.
2. Key Laboratory of Biomimetic Robots and Systems (Beijing Institute of Technology), Ministry of Education, Beijing 100081, China.

* Corresponding author. Email: shiqing@bit.edu.cn (Q. Shi.)

**1. Proof of the expected interaction information entropy being the function of animal motion description variables.**

Given that both the states and the actions are the sequences of animal joints movements, they can be described as

$$\begin{aligned} \begin{matrix} s=F_{s}\left( \Theta\right)=\left( \Theta_{1},\Theta_{2},\cdots,\Theta_{N} \right) \\ a=F_{a}\left( \Theta\right)=\left( \Theta_{1},\Theta_{2},\cdots,\Theta_{n} \right) \end{matrix}\#\left( S1 \right) \end{aligned}$$

where $N$ and $n$ represents the length of the states and actions respectively. The expected interaction information entropy can be written as

$$EI\left( s,a \right)= EF\left( s^{t},s^{t+1},a^{t} \right)$$

$$=EF\left( F_{s}^{t},F_{s}^{t+1},F_{a}^{t} \right)$$

$$=EF\left( F_{s}^{t},F_{s}^{t}p\left( s^{t+1} | s^{t},a^{t} \right),F_{a}^{t} \right)$$

$$\begin{aligned} =F\left( EF_{s}^{t},{EF}_{s}^{t}p\left( s^{t+1} | s^{t},a^{t} \right),{EF}_{a}^{t} \right)\#\left( S2 \right) \end{aligned}$$

$$=F\left( F_{s}^{t},F_{s}^{t}Ep\left( s^{t+1} | s^{t},a^{t} \right),F_{a}^{t} \right)$$

$$=F\left( F_{s}^{t},F_{s}^{t}\sum_{s\in\mathcal{S}} p\left( s | s^{t},a^{t} \right),F_{a}^{t} \right)$$

$$=G\left( \Theta^{t} \right)$$

where $G$ represents an unclear function of the animal motion description variables $\Theta^{t}$. The Equation S2 proved that the expected value of $I$ is independent with the future motions.

**2. The accumulated reward of the state.**

The mathematical formulation of the tabular TD can be written as

$$\begin{aligned} V_{\pi}\left( s \right)=E_{a\sim\pi}\left\{ \sum_{t=0}^{\infty} \gamma\tau R_{t}\left( s_{t},a_{t} \right) | s_{0}=s \right\}\#\left( S3 \right) \end{aligned}$$

In Equation S3, $V\_\pi$ satisfies the Hamilton Jacobi Bellman Equation. Therefore it can be written in

$$\begin{aligned} V_{\pi}\left( s \right)=E_{\pi}\left[ R\left( s_{0} \right)+\gamma V_{\pi}\left( s_{1} \right) | s_{0} \right]\#\left( S4 \right) \end{aligned}$$

where $R(s_{0})$ is the accumulate reward of the initial state $s_{0}$. From the above equation, it can be proved that $R\left( s_{0} \right)+\gamma V_{\pi}\left( s_{1} \right)$ is an unbiased estimate for $V_{\pi}\left( s \right)$. The policy then can be updated each time it generates new actions in the state $s$ with the accumulate reward as Equation 6.
